# Effect of intubation and mechanical ventilation on exhaled nitric oxide in preterm infants with and without bronchopulmonary dysplasia measured at a median postmenstrual age of 49 weeks

**DOI:** 10.1186/1756-0500-7-389

**Published:** 2014-06-24

**Authors:** Gerd Schmalisch, Silke Wilitzki, Hendrik S Fischer, Christoph Bührer

**Affiliations:** 1 Department of Neonatology, Charité University Medicine, Charitéplatz 1, D - 10117 Berlin, Germany

**Keywords:** Prematurity, Exhaled nitric oxide, Mechanical ventilation, Bronchopulmonary dysplasia, Lung function test, Neonate

## Abstract

**Background:**

Exhaled nitric oxide (eNO) is a marker of established airway inflammation in adults and children, but conflicting results have been reported in preterm infants when postnatal eNO is measured during tidal breathing. This study investigated the extent to which intubation and mechanical ventilation (MV) affect eNO and NO production (V’_NO_) in preterm infants with and without bronchopulmonary dysplasia (BPD).

**Patients and methods:**

A total of 176 very low birth weight (VLBW) infants (birth weight <1500 g), including 74 (42%) with and 102 (58%) without BPD, were examined at a median postmenstrual age of 49 weeks. Of the 176 infants, 84 (48%) did not require MV, 47 (27%) required MV for <7 days and 45 (26%) required MV for ≥7 days. Exhaled NO and tidal breathing parameters were measured in sleeping infants during tidal breathing, respiratory mechanics were assessed by occlusion tests, and arterialized capillary blood gas was analyzed.

**Results:**

eNO was significantly correlated with tidal breathing parameters, while V’_NO_ was correlated with growth parameters, including age and body length (p < 0.001 each). Infants who were intubated and received MV for <7 days had significantly lower eNO (p < 0.01) and V’_NO_ (p < 0.01) than non-ventilated infants. In contrast, eNO and V’_NO_ did not differ significantly in non-ventilated infants and those receiving MV for ≥7 days. Multivariate analysis showed that independent on the duration of MV eNO (p = 0.003) and V’_NO_ (p = 0.018) were significantly increased in BPD infants comparable with the effects of intubation and MV on eNO (p = 0.002) and V’_NO_ (p = 0.017).

**Conclusions:**

Preterm infants with BPD show only weak postnatal increases in eNO and V’_NO_, but these changes may be obscured by the distinct influences of breathing pattern and invasive respiratory support. This limits the diagnostic value of postnatal eNO measurements in the follow-up of BPD infants.

## Background

Chronic lung disease (CLD) of prematurity, also called bronchopulmonary dysplasia (BPD), is the most common long-term pulmonary disease after preterm birth and neonatal intensive care [1] with consequences for later lung function [2-4]. Although inconsistent findings have been reported, there is a consensus that prematurity *per se* may make a more important contribution to impaired lung function than BPD [5-7]. Moreover, in a recent study [8] we have shown that prematurity and neonatal lung disease, followed by mechanical ventilation (MV), are independently associated with impaired postnatal lung function.

Bronchial hyperreactivity is common in former preterm infants with BPD and is likely due to airway damage in early infancy caused by intubation and MV [9,10]. Besides arresting lung maturation, lung inflammation is a major contributor to the pathogenesis of BPD. Since lung inflammation may be caused primarily by oxygen and/or MV, there is increased interest in postnatal measurements of exhaled nitric oxide (eNO), a marker of eosinophilic airway inflammation in adults and older children [11]. However, studies of postnatal eNO in BPD infants have yielded conflicting results, with some showing increased eNO in BPD infants [12,13] and others showing no differences in infants with and without BPD [14,15]. Moreover, unchanged or even decreased eNO has been observed when former BPD infants reached school age [16-19].

A possible explanation for these conflicting results may be differences in patient populations, as well as differences in equipment and measuring conditions [20]. NO concentration in exhaled air is highly air flow-dependent and originates primarily in the nose and the paranasal sinuses, with some also originating in the lower part of the respiratory tract [21,22]. Therefore, in adults and older children the measurement conditions have been standardized [23]. Online-measurements of eNO in spontaneously breathing newborns, however, can only be performed during tidal breathing, with a face mask that assesses mixed air from the nose and upper airway; hence such measurements in newborns may be affected by variations in breathing pattern and often an eNO plateau is missing in the exhaled air. We hypothesized that, in spontaneously breathing neonates, the different determinants of eNO may account for differences in eNO between BPD and non-BPD infants. This study was therefore designed to investigate the effects of breathing pattern and invasive respiratory support during the first days of life on differences in eNO between non-BPD and BPD infants at 46–54 postmenstrual weeks.

## Methods

### Subjects

This retrospective study examined eNO concentrations in 176 very low birth weight (VLBW) infants, defined as birth weights <1500 g. Infants were assessed between June 2009 and June 2013 during lung function testing (LFT) as part of our routine follow-up care of preterm infants. At the time of measurement, the median age of the 176 infants was 49 postmenstrual weeks. Infants with congenital diaphragmatic hernia, congenital heart disease, neuromuscular disease, or thoracic wall deformities were excluded. Included infants were classified into three groups according to the degree of respiratory support during the first days of life in the same manner as in a former follow-up study [8]: 1) those without or with only a mild respiratory insufficiency who could be treated by non-invasive techniques (non-ventilated infants); 2) those requiring intubation and MV for <7 days; and 3) those requiring MV for ≥7 days. INSURE (INtubation, SURfactant, Extubation) [24] was not considered MV. Subjects were also classified as with or without BPD, with BPD defined as a requirement for supplemental oxygen at a postmenstrual age of 36 weeks.

All parents provided written informed consent before each LFT, and the Institutional Data Safety Committee approved this study.

### eNO measurements

eNO was measured during routine LFT in clinically stable children who had no respiratory infections during the 3 weeks preceding the tests. Prior to testing, body weight was measured to the nearest 10 g (Seca, Hamburg, Germany) and body length (crown to heel) to the nearest 5 mm.

After a temperature stabilization period of at least 30 min, all devices were calibrated before measurement according to the manufacturers’ guidelines. Sleeping infants were measured in a supine position with the neck in a neutral position and supported by a neck roll. Sleep was induced 15–30 min before LFT by oral administration of chloral hydrate (50 mg∙kg^-1^), since sedation was necessary for more complex LFT [8]. A compliant silicon mask (Infant mask, size 1; Vital Signs Inc., Totowa, NJ, USA) was tightly placed over the nose and mouth of each subject.

An NO filter ensured that all infants inhaled NO-free air. eNO and tidal breathing were measured using the EXHALYZER D (EcoMedics AG, Duernten, Switzerland), which measures lung function in infants, and the fast chemiluminescence NO-analyzer CLD 88 (EcoMedics). Since eNO is strongly flow dependent, the EXHALYZER D also calculates NO production (V’_NO_) to consider different flow conditions [15]. eNO was measured during the third quartile of expiration, since it showed the lowest breath-to-breath variability [25,26]. V’_NO_ was calculated by multiplying V’ by steady-state eNO (V’_NO_= V’∙eNO).

Simultaneously, the following tidal breathing parameters were recorded: tidal volume (V_T_), respiratory rate (RR), minute ventilation (V’_E_), expiratory time (t_e_), the ratio of time to peak tidal expiratory flow to expiratory time (t_ptef_/t_e_), peak tidal inspiratory flow (PTIF), peak tidal expiratory flow (PTEF), mean tidal inspiratory flow (MTIF) and mean tidal expiratory flow (MTEF). Shortly afterwards, respiratory mechanics (respiratory compliance (C_rs_) and respiratory resistance (R_rs_)) were measured by the single occlusion test using the same face mask and a balloon shutter to achieve brief airway occlusion at end-inspiration, as described [27]. Between 5 and 15 occlusions were performed and the mean of 5 valid measurements of C_rs_ and R_rs_ reported. All measurements were performed using the MasterScreen™ BabyBody (CareFusion, Höchberg, Germany). All tidal breathing flow and volume parameters and C_rs_ were divided by body weight on the day of measurement to reduce inter-subject variability.

An arterialized capillary blood gas sample was taken at the end of the LFT (ABL800 FLEX, Radiometer, Denmark). Heart rate and oxygen saturation were monitored continuously during the LFT by a pulse oximeter (N-200; Nellcor, Hayward, California, USA).

### Statistical methods

Patient characteristics are reported as rates or as medians and interquartile ranges (IQR). Gender-specific birth weight *z*-scores were calculated using published national reference data [28]. Data were compared among the three ventilation groups by the chi-square test, Fisher’s exact test or the Kruskal-Wallis rank test, as appropriate. Lung function parameters of the three ventilation groups are reported as median (IQR) and the differences among the three groups were tested by the Kruskal-Wallis rank test with the Mann–Whitney test as post-hoc test. The effect of gender, antenatal steroids and surfactant treatment on eNO and V’_NO_ was tested using the Kruskal-Wallis test. The relationships of eNO and V’_NO_ with the anthropometric and lung function parameters were assessed by Spearman rank order correlation coefficients with Bonferroni correction for multiple correlations. The effect of BPD and mechanical ventilation on eNO and V’_NO_ and their interactions were investigated by two way analysis of variance (ANOVA), with anthropometric and tidal breathing parameters as covariates. All statistical analyses were performed using Statgraphics Centurion® software (Version 16.0, Statpoint Inc., Herndon, VA, USA), with a p-value < 0.05 considered statistically significant.

## Results

### Patient characteristics

eNO and V’_NO_ were measured in 176 VLBW infants at a median (IQR) postmenstrual age of 49.0 (45.6 - 54.4) weeks. Of these infants, 74 (42%) formerly had BPD, and 92 (52%) had a severe respiratory insufficiency that required intubation and intermittent MV with a nasally inserted endotracheal tube for at least 24 h. Of the ventilated infants, 47 (27%) required MV for <7 days and 45 (26%) for ≥7 days. The median (IQR) duration of MV was 7 (1–14) days, and was significantly longer for BPD than for non-BPD infants (10 (3 – 27) days vs. 2.5 (1 – 7.5) days, p < 0.001).

Table 1 compares patient characteristics relative to the duration of MV. At the time of measurement, there were no statistically significant between-group differences in chronological age, postmenstrual age, body weight, and body length. Infants requiring MV were significantly (p < 0.001) more immature, had a lower birth weight and a higher incidence of surfactant administration. However, there were no statistically significant differences of birth weight *z*-scores between the ventilation groups. The incidence of BPD was higher in ventilated than in non-ventilated infants. Only the incidence of fetal lung maturation was similar in the ventilation groups.

**Table 1:** Patient characteristics in the neonatal period and at the time of measurement according to the duration of mechanical ventilation, presented as median and interquartile range (in brackets) or N (%)

	**Not ventilated infants**	**Intubated and ventilated < 7days**	**Intubated and ventilated ≥ 7days**	**p-value**
	**N = 84**	**N = 47**	**N = 45**	
*Neonatal period*				
Gestational age (weeks)	29	27	26	**<0.001**
	(28–30)	(26 – 28)	(25 – 27)	
Birth weight (g)	1145	930	803	**<0.001**
	(890–1340)	(740–1230)	(580 – 950)	
Birth weight *z*-score	-0.33	-0.14	-0.44	0.305
	(-0.99 - 0.33)	(-0.88 - 0.56)	(-1.18 - 0.14)	
Antenatal steroids^1)^	59/81 (73%)	33/42 (79%)	41/45 (91%)	0.053
Surfactant administration^1)^	41/83 (49%)	43/43 (100%)	42/45 (93%)	**<0.001**
BPD	14 (17%)	25 (53%)	35 (78%)	**<0.001**
*At day of measurement*				
Age (days)	149	142	158	0.105
	(116–204)	(116 – 166)	(143 – 181)	
Postmenstrual age (weeks)	49.9	48.4	48.3	0.114
	(46.9 – 58.0)	(44.0 – 51.3)	(46.4 – 53.4)	
Body length (cm)	57.75	57	56.0	0.155
	(55.0 – 62.3)	(53 – 60.0)	(54–60.0)	
Body weight (g)	5052.5	4900	4815	0.210
	(4315–6205)	(4200 – 5860)	(4100 – 5580)	

^1)^Reduced total number due to incomplete data of patients examined by LFT.

Data represent median and interquartile range (in brackets) or N (%). Statistically significant p-values are printed in bold.

### Factors associated with eNO and V’NO

There were no statistically significant associations between eNO and V’_NO_ levels and gender, use of antenatal steroids, or surfactant treatment. Despite the strong correlation between eNO and V’_NO_(r = 0.778, p < 0.001), factors associated with them differed markedly. Table 2 shows the correlation of eNO and V’_NO_ with age, anthropometric parameters at birth and at the time of measurement and lung function parameters. After Bonferroni correction for multiple correlations, eNO showed statistically significant correlations only with tidal breathing parameters whereas V’_NO_ correlated significantly with growth parameters (age, postmenstrual age, body length), respiratory rate and duration of expiration. Gestational age, birth weight and birth weight *z*-scores failed to display a statistically significant correlation with eNO and V’_NO._

**Table 2:** **Spearman rank order coefficients of eNO and V’**_**NO**_**with patient characteristics at birth and at day of measurements and lung function parameters**

	**eNO (ppb)**		**V’NO (nL/s)**	
	***R***_***s***_	**p-value**	***R***_***s***_	**p-value**
*Patient data at day of birth*				
Gestational age (weeks)	-0.103	0.176	-0.058	0.447
Birth weight (g)	-0.105	0.167	-0.045	0.555
Birth weight Z-score	0.033	0.666	0.049	0.515
*Patient data at day of measurement*				
Age (days)	0.031	0.682	**0.264**	**<0.001**
PMA (weeks)	-0.007	0.926	**0.237**	**0.002**
Body weight (g)	-0.033	0.655	0.210	0.006
Body length (cm)	0.002	0.985	**0.250**	**0.001**
*Tidal breathing*				
V_T_ (mL/kg)	0.011	0.883	0.151	0.047
RR (1/min)	-0.216	0.004	**-0.266**	**<0.001**
V’_E_ (mL/min/kg)	-0.228	0.003	-0.170	0.025
t_ptef_/t_e_ (%)	-0.110	0.148	-0.113	0.135
t_e_ (s)	**0.274**	**<0.001**	**0.281**	**<0.001**
PTIF (mL/s/kg)	-0.062	0.415	-0.083	0.272
PTEF (mL/s/kg)	**-0.230**	**0.002**	-0.163	0.032
MTIF (mL/s/kg)	-0.121	0.112	-0.107	0.158
MTEF (mL/s/kg)	**-0.274**	**<0.001**	-0.192	0.011
*Respiratory mechanics*				
C_rs_ (mL/kPa/kg)	-0.027	0.725	0.058	0.450
R_rs_ (kPa/L/s)	0.078	0.318	-0.162	0.037
*Blood gas analysis*				
paO_2_ (mmHg)	-0.059	0.434	0.147	0.053
paCO_2_ (mmHg)	0.158	0.037	-0.037	0.628

*R*_*s*_, Spearman rank order correlation coefficient, statistically significant values after Bonferroni correction (p < 0.0025) are shown in boldface.

Abbreviations: PMA - postmenstrual age, V_T_ - tidal volume, RR - respiratory rate, V’_E_- minute ventilation, t_ptef_/t_e_ - ratio of time to peak tidal expiratory flow to expiratory time, t_e_ - expiratory time, PTIF - peak tidal inspiratory flow, PTEF - peak tidal expiratory flow, MTIF - mean tidal inspiratory flow, MTEF - mean tidal expiratory flow, C_rs_ - respiratory compliance, R_rs_ - respiratory resistance.

The correlations between eNO and mean and peak (MTEF, PTEF) tidal expiratory flow were negative. Thus, as expiratory flow increased, the eNO concentration decreased. In contrast, eNO increased with increasing expiratory time.

NO production was positively correlated with age and anthropometric parameters, indicating that V’_NO_ increased with growth. There were no statistically significant correlations between V’_NO_ and tidal breathing parameters, except for a significant negative correlation between V’_NO_ and respiratory rate and a strong positive correlation with the expiratory time (both p < 0.001).

### Effect of intubation and mechanical ventilation on eNO and V’NO

Table 3 shows the measured lung function parameters in the three patient groups classified by duration of MV. Of the tidal breathing parameters, only tidal volume showed a slight decrease (p = 0.034) and respiratory rate showed a slight increase in ventilated patients (p = 0.045). The compliance of the ventilated infants was also reduced, but differed significantly only between non-ventilated infants and infants mechanically ventilated for ≥7 days. There was no significant difference in respiratory resistance between the three patient groups. Blood gases, however, differed significantly among the three MV groups, with pO_2_ decreasing and pCO_2_ increasing significantly as the duration of MV increased (both p < 0.001).

**Table 3:** Lung function test results in VLBW infants according to the duration of mechanical ventilation

	**Not ventilated infants**	**Intubated and ventilated < 7days**	**Intubated and ventilated ≥ 7days**	**p-value**
	**N = 84**	**N = 47**	**N = 45**	
*Tidal breathing*				
V_T_ (mL/kg)	8.61	8.01	7.68**	**0.034**
	(7.37 - 9.80)	(6.70 - 9.67)	**(6.96 - 8.64)**	
RR (1/min)	40.9	44.8	44.4*	**0.045**
	(36.0 - 47.1)	(35.5 - 56.1)	**(38.0 - 55.7)**	
V’_E_ (mL/min/kg)	346.2	373.0	353.0	0.452
	(297.8 - 398.4)	(311.2 - 407.3)	(299.0 - 402.7)	
t_ptef_/t_e_ (%)	20.7%	22.1%	21.1%	0.898
	(17.4% - 26.2%)	(16.3% - 27.0%)	(17.1% - 27.2%)	
t_e_ (s)	0.857	0.770	0.753*	**0.025**
	(0.720 - 0.962)	(0.593 - 0.990)	**(0.577 - 0.861)**	
PTIF (mL/s/kg)	18.9	19.6	18.0	0.351
	(15.4 - 21.3)	(16.6 - 22.6)	(16.4 - 20.7)	
PTEF (mL/s/kg)	17.1	18.0	17.9	0.150
	(14.6 - 19.4)	(15.2 - 22.0)	(14.5 - 20.9)	
MTIF (mL/s/kg)	13.4	14.0	13.1	0.642
	(11.5 - 15.4)	(12.0 - 16.3)	(11.8 - 15.7)	
MTEF (mL/s/kg)	10.0	11.1	10.4	0.375
	(8.6 - 12.0)	(9.3 - 12.5)	(8.9 - 13.0)	
*Respiratory mechanics*				
C_rs_ (mL/kPa/kg)	10.6	9.2	8.9***	**0.002**
	(9.0 - 11.0)	(7.6 - 11.6)	**(6.7 - 10.5)**	
R_rs_ (kPa/L/s)	5.96	6.55	6.15	0.094
	(4.61 - 7.30)	(5.07 - 8.62)	(5.17 - 7.86)	
*Exhalation gas analysis*				
eNO (ppb)	10.0	6.6**	10.4	**0.005**
	(6.3 - 13.6)	**(4.2 - 10.7)**	(7.2 - 13.8)	
V’_NO_ (nL/s)	0.50	0.39**	0.54	**0.005**
	(0.34 - 0.72)	**(0.24 - 0.54)**	(0.37 - 0.66)	
*Blood gas analysis*				
paO_2_ (mmHg)	73.3	66.6*	65.6***	**< 0.001**
	(65.5 - 83.0)	**(58.6 - 78.0)**	**(58.5 - 70.9)**	
paCO_2_ (mmHg)	39.9	42.2*	44.6***	**< 0.001**
	(37.7 - 43.7)	**(39.1 - 45.8)**	**(41.1 - 47.3)**	

*p < 0.05, ** p < 0.01, *** p < 0.001 when compared to not ventilated infants. Data represent median (interquartile range). Statistically significant values are printed in bold.

Intubation and mechanical ventilation had different effects on eNO and V’_NO_. The largest differences were seen in infants ventilated <7 days, where eNO and V’_NO_ were significantly reduced (p < 0.01). In contrast, results were similar in long-term ventilated and non-ventilated infants.

### Effects of BPD on eNO and V’NO

**Figure 1:**
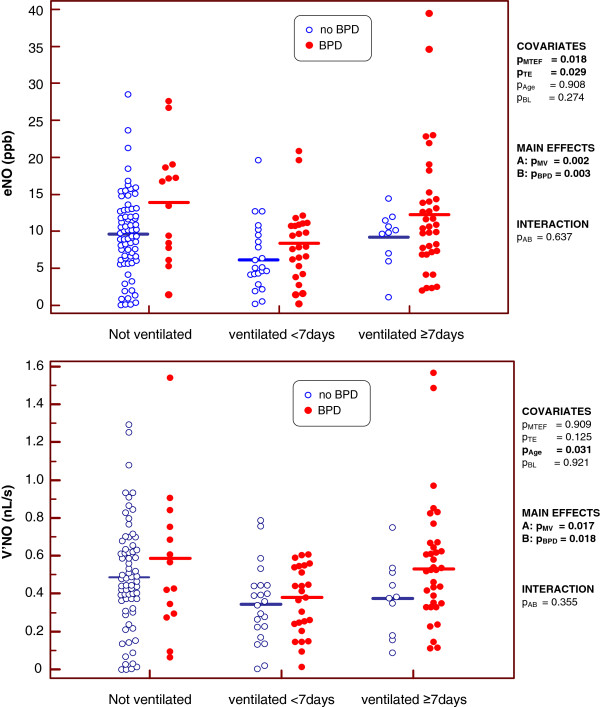
**Effect of BPD and the duration of mechanical ventilation on the exhaled NO (top) and the NO production (bottom).** The p-values show the extent of statistical significance of the covariates mean tidal expiratory flow (p_MTEF_), expiratory time (p_TE_), age (p_Age_) and body length (p_BL_); those of the main effects by mechanical ventilation (p_MV_) and BPD (p_BPD_); and that of the interaction (pAB) of both factors. Statistically significant values are shown in bold.

To consider the dependency of eNO and V’_NO_ on MV duration, the effect of BPD on both parameters was investigated by two way ANOVA, after adjustments for age, body length, expiratory time t_e_ and MTEF. As shown in Figure 1, both BPD and the duration of MV had significant effects on eNO, with a lesser effect on V’_NO_. Despite different levels in all ventilation groups, mean eNO and V’_NO_ were higher in BPD than in non-BPD infants.

Multivariate analysis showed that the tidal breathing parameters t_e_ and MTEF were significantly associated with eNO, whereas anthropometric measures were not. V’_NO_ was significantly affected only by chronological age_._ BPD and MV had no interactive effects on eNO and V’_NO_ (all p-values >0.1).

## Discussion

The results presented here demonstrate that, in very premature infants, BPD and intubation and MV due to severe respiratory insufficiency affect eNO and V’_NO_ independently and in different directions. The measured median (IQR) eNO in non-ventilated infants without BPD (10.0 (6.3 - 13.6) ppb) was slightly lower than reference values published by Fuchs et al. [29] (14 (10.7–17.4) ppb) using the same equipment measured in unsedated term-born healthy infants at 5 weeks of age. In our study, infants with previous BPD showed weak but statistically significant increases in eNO and V’_NO,_ whereas intubation and MV for <7 days decreased both parameters and did so to a greater degree. Thus, eNO may be lower in a short term ventilated BPD infant than in a non-ventilated infant without BPD. This hampers the clinical interpretation of postnatal eNO and V’_NO_ and can lead to conflicting results, as shown in recent studies measuring postnatal eNO in infants with and without chronic lung disease (CLD) or BPD.

Leipala et al. [13] examined infants with chronic lung disease (CLD) using tidal breathing measurements with a face mask at 36–45 weeks post-conception. They found higher peak nasal (p = 0.011) and facemask (p = 0.034) NO levels in CLD than in non-CLD infants, in agreement with our findings of weakly but significantly raised eNO in BPD infants. May et al. [12] found also elevated eNO in very premature intubated infants during MV on day 28 after birth, particularly in infants developing moderate or severe BPD [12]. However, Wiliams et al. [30] have shown that eNO during the first 4 weeks of life did not differ significantly in groups of infants with and without CLD, indicating that, in contrast to conventional LFT parameters (e.g. compliance, functional residual capacity), eNO measurements during the first weeks of life are not useful for predicting CLD and that eNO levels may be raised only in infants with established BPD and ongoing inflammation [30]. In contrast to the previous studies Roiha et al. [15], measuring eNO and V’no during tidal breathing in 39 unsedated pre-term infants with CLD (mean gestational age 27.3 weeks), found no statistically significant differences to term infants (39.9 weeks). Following adjustment of eNO and V’_NO_ for ventilatory parameters, V’_NO_ was slightly decreased in CLD infants (p = 0.024). In that study the median (range) duration of intubation and MV was 1 (0–63) days, indicating that most CLD infants received MV for only a short time. It remains unclear whether this may have influenced the results in the same way as in the present study.

It also remains unclear why eNO and V’_NO_ are reduced in short-term ventilated preterm infants. Nasal intubation and MV may induce ciliary dyskinesia, characterized by reduced eNO [22,31,32]. This would be on accordance with the findings of Baraldi et al. [19]. In this study eNO was reduced in school aged children who formerly had CLD [19]. They speculated that an impaired NO synthesis and/or diffusion in the airways could be a sequela of epithelial damage during the early phases of BPD. Filiponne et al. [16] have shown that children born prematurely with and without BPD have increase oxidative stress compared to children born at term despite normal eNO levels, pointing to other inflammatory pathways that cannot be picked up by eNO measurements. Ricciardlo et al. [33] investigated determinants of eNO in childhood atopic asthma. They found that neonatal respiratory distress has been associated with low eNO in children with atopic asthma [33]. The higher eNO and V’_NO_ in long-term (≥7 days) than in short-term (<7 days) ventilated infants may have been caused by established ongoing inflammation secondary to prolonged intubation and mechanical ventilation as well as exposure to supplemental oxygen [34].

We previously showed that, in preterm infants, intubation and MV were mainly associated with increased tissue and airway resistances, reduced effective lung volume and impaired blood gases [8]. However, we could not determine whether these changes were caused by intubation and MV or by the underlying respiratory disease. In the present study, we showed that intubation and MV due to neonatal lung diseases also affected eNO and V’_NO,_ although these effects depended on the duration of MV. Because infants with acute respiratory tract infection were excluded from the study, these effects on eNO were negligible [29]. Except for tidal breathing and anthropometric parameters, we could not find further associations with eNO and V’_NO_ levels, including data at birth. Unfortunately, in this study we had no verified access to data on maternal atopic disease [35], prenatal tobacco exposure [25,26] or traffic-related air pollution [36,37] and thus could not investigate the impact of these confounders on exhaled NO.

Ideally, eNO should be measured while the subject is breathing out at a constant expiratory flow rate to obtain an eNO plateau. Adults and older children exhale at a constant flow (V’) against a resistance, resulting in a plateau in the eNO signal [38]. However, this is not possible during postnatal tidal breathing. eNO measured during the third quartile of expiration should mimic the conditions of flow standardization in adults [25]. However, a higher respiratory rate and a shorter expiratory time increase the likelihood that an eNO plateau will not be achieved, a phenomenon known from capnographic measurements in small lungs [39], especially at appearance of air leaks [40]. The lack of a plateau in exhaled NO can lead to underestimations of eNO and V’_NO_ and may contribute to the negative correlations between these parameters and respiratory rate and the positive correlations with expiratory time.

Therefore, a numerical adjustment of the measured eNO and V’_NO_ is recommended in comparative studies [12] considering the differences in development and breathing pattern [41,42]. Any such numerical adjustment, however, can only be useful if eNO and V’_NO_ are measured equally accurately across the range of respiratory rates of the sample. Using the measurement equipment available today, the reliability of eNO and V’_NO_ measurements in preterm infants is limited by the response time of the NO-analyzer. The strong decrease of V’_NO_ with increasing respiratory rate (Table 2) may be caused by this technical problem arising when the exhalation time is short. These limitations may also contribute to the conflicting results of the published studies.

This study has several strengths and limitations. The main strengths include the use of a large sample size classified by duration of MV and the use of the same investigator, equipment, and protocol for all patients, since measurements of lung function in infants of this age are highly dependent on device and protocol. The study limitations include its retrospective design and the lack of a control group of healthy infants [43]. Because this study was retrospective, important maternal (e.g., smoking, atopy, asthma, education), neonatal (e.g., birth characteristics, resuscitation management, postnatal steroids, infections) and environmental parameters (e.g., smoking exposure, air pollution) which may affect the postnatal lung development [44,45] were unavailable. Further clinical data (e.g., the duration of non-invasive respiratory support or oxygen treatment) may help to understand the pathophysiology underlying this condition. Furthermore, the extreme skewness in the patient distribution (there were only 10 long-term ventilated infants without BPD) limits the number of parameters for multivariate modeling.

## Conclusion

Online tidal breathing measurements of eNO and V’_NO_ via a face mask are a simple and noninvasive technique for assessing postnatal lung function. The inability to standardize measurement conditions during tidal breathing can result in a high dependency of measured parameters on breathing patterns. Although mean eNO and V’_NO_ are increased in BPD infants, these increases are only weak and may be obscured by other factors affecting eNO during tidal breathing. Thus the interpretation of individual measurements is difficult due to opposing influencing factors, making eNO and V’_NO_ unlikely to be helpful in following up BPD infants during the first months of life.

## Abbreviations

BPD: Bronchopulmonary dysplasia; CLD: Chronic lung disease; C_rs_: Respiratory compliance; eNO: Fractional exhaled nitric oxide.; IQR: Inter-quartile range; LFT: Lung function testing; MTEF: Mean tidal expiratory flow; MTIF: Mean tidal inspiratory flow; MV: Mechanical ventilation; PMA: Postmenstrual age; PTEF: Peak tidal expiratory flow; PTIF: Peak tidal inspiratory flow; RR: Respiratory rate; R_rs_: Respiratory resistance; *R*_*s*_: Spearman rank order correlation coefficient; TB: Tidal breathing; t_e_: Expiratory time; t_PTEF_/t_E_: Time to peak tidal expiratory flow related to the expiratory time; V’: Air flow; V’_E_: Minute ventilation; VLBW: Very low birth weight (<1500 g); V’_NO_: Nitric oxide production; V_T_: Tidal volume.

## Competing interests

None of the authors has a financial relationship with a commercial entity that has an interest in the subject of this manuscript.

## Authors’ contribution

GS had primary responsibility for study design, data analysis and writing of the manuscript. SW carried out all lung function measurements and GS performed statistical analysis. HF and CB participated in the development of the study and contributed to the writing of the manuscript. All authors read and approved the final manuscript.
